# The Interplay Between Summer Meals, Food Insecurity, and Diet Quality Among Low-Income Children in Maryland, USA: A Multiphase Cross-Sectional Study

**DOI:** 10.3390/nu17132055

**Published:** 2025-06-20

**Authors:** Yuyi Chen, Erin R. Hager, Julia Gross, Susan M. Gross

**Affiliations:** 1 Graduate Group in Nutritional Biology, University of California, Davis, CA 95616, USA; 2 Department of Population, Family and Reproductive Health, Johns Hopkins Bloomberg School of Public Health, Baltimore, MD 21205, USA; ehager1@jhmi.edu; 3 Maryland Hunger Solutions, Baltimore, MD 21211, USA; jgross@mdhungersolutions.org

**Keywords:** summer meals, child nutrition, food insecurity, diet quality, out-of-school-time nutrition, federal nutrition programs, Summer Food Service Program, nutrition program evaluation

## Abstract

**Background:** Food insecurity and poor diet quality disproportionately affect U.S. children from low-income households, with summer school closures exacerbating risks. Federally funded programs like the Summer Food Service Program (SFSP) and SUN Bucks (Summer EBT) aim to address these challenges, yet evidence of their post-pandemic dietary impact remains limited. **Objectives:** This study examines the relationship between policy innovations, summer meal participation, food insecurity, and diet quality among children from low-income households in Prince George’s County, Maryland. **Methods:** A cross-sectional design analyzed data from 158 households in Prince George’s County Public Schools across two waves (early fall 2022 and 2023). Validated tools (USDA’s Six-Item Short Form and Dietary Screener Questionnaire) assessed food security and diet quality. Sociodemographic factors, program participation, and dietary deviations from the 2020–2025 Dietary Guidelines were analyzed. Multivariate logistic regression identified determinants of poor diet quality (≥2 guideline deviations), adjusting for ethnicity, age, and housing stability. **Results:** Only 32.28% of eligible households participated in summer meal programs, with non-participation driven by lack of awareness (53.68%) and transportation barriers (11.58%). Significant dietary gaps included inadequate whole grain intake (0.8 vs. 3.0 servings/day) and excessive added sugars (14% of daily calories). Summer meal participation was associated with reduced odds of poor diet quality (OR = 0.23, *p* = 0.030), while older age (OR = 52.97, *p* < 0.001) and very low food security (OR = 8.42, *p* = 0.036) increased risk. Hispanic ethnicity had lower odds (OR = 0.17, *p* = 0.019) despite higher baseline food insecurity. **Conclusions**: Summer meal participation was associated with improved dietary outcomes but faced systemic participation barriers. Findings support policy reforms, such as multilingual outreach and mobile meal distribution, to address identified gaps.

## 1. Introduction

Food insecurity and poor diet quality are critical public health challenges in the United States, disproportionately affecting children from low-income and minority households [1,2]. Food insecurity, defined as limited or uncertain access to adequate food, has been linked to developmental delays, poor academic performance, and chronic disease risk in children [3]. Federally funded programs—including the National School Lunch Program (NSLP), the School Breakfast Program (SBP), and Community Eligibility Provision (CEP)—provide school meals in high-poverty areas without household applications, while the Supplemental Nutrition Assistance Program (SNAP) and the Special Supplemental Nutrition Program for Women, Infants, and Children (WIC) provides free nutritional assistance to low-income families [4,5]. Child food insecurity intensifies during summer due to school closures, which disrupt access to NSLP and SBP [6]. To bridge this gap, the Summer Food Service Program (SFSP) provides free meals, while the Summer Electronic Benefit Transfer (SUN Bucks)—a permanent USDA program established in 2023—offers electronic grocery benefits [7,8,9]. Despite their essential role, the effectiveness of these nutrition programs in improving children’s diet quality and food security remains inadequately studied, particularly in the context of broader systemic changes during the COVID-19 pandemic.

The COVID-19 pandemic exacerbated preexisting food insecurity while school closures disrupted access to school meals [10,11]. In response, the Families First Coronavirus Response Act granted the USDA the authority to issue nationwide waivers for school meal programs, allowing them to operate like summer meal programs, such as the SFSP and the NSLP Seamless Summer Option (SSO). These pandemic waivers enabled (1) extended operation beyond traditional summer months to ensure year-round meal access during closures, (2) expanded sponsor eligibility to non-traditional providers, such as community centers and food banks, to broaden distribution networks, (3) adaptive meal distribution methods, such as curbside pickup, multiple-meal bundling, and parent or guardian pickup, to minimize viral exposure, and (4) relaxed nutritional standards to address supply chain disruptions while maintaining federal financial reimbursement eligibility [12,13,14]. These adjustments carried into the summer of 2022, expanding access to summer meals for children from underserved communities [15,16].

Parallel to these temporary solutions, Direct Certification through Medicaid (DC-M) was piloted in Maryland during the 2022–2023 school year to address structural barriers to food assistance programs [17]. DC-M automates eligibility verification for free or reduced-price meals using Medicaid data, which has the potential to reduce administrative burdens while encouraging program participation [18]. The expiration of pandemic-related waivers and the implementation of DC-M coincided in 2022, presenting a unique opportunity to examine the relationships between summer meal participation, food insecurity, and dietary patterns among children in low-income households during periods of policy innovation.

The existing literature primarily examines the effect of policy changes on program reach. Previous studies have demonstrated that pandemic-related waivers increased organizational sponsorships by 28% [19] and enabled service expansion to low-income areas through mobile distribution systems [20]. The enhanced accessibility of summer meal programs encouraged household participation, resulting in a 34% increase in total meals served between 2019 and 2021 [19,20,21,22], with low-income areas accounting for 40% of participation gains [20,21]. However, the waiver’s expiration in 2022 disrupted services due to higher operational costs, particularly for smaller sponsors that relied on mobile distribution systems [15,23]. Rural communities experienced disproportionate service reductions, with a 22% decline in meal sites within six months of waiver expiration [15]. DC-M mitigated some losses, resulting in an 8% increase in NSLP enrollment, although rural–urban disparities in implementation efficacy persisted [19].

While policy innovations during the pandemic successfully increased program participation rates [19], a critical gap persists in evaluating their nutritional effectiveness—the central metric of program success. Despite the fundamental role of diet quality in shaping child health and development [24], there is a dearth of studies that systematically assess dietary outcomes during this period of public health crisis and policy changes. This gap stems partly from methodological challenges in longitudinal dietary tracking during the pandemic, resulting in a disconnect between program reach and nutritional outcomes. Such a limitation hinders evidence-based policy adjustments to optimize child nutrition.

This study addresses this critical gap by examining the dynamic relationship between policy innovations, summer meal participation, food insecurity, and diet quality among low-income children in Prince George’s County, Maryland, before and after the expiration of pandemic-related waivers and DC-M implementation in 2022. Findings from this study may inform policy and programmatic decisions to improve child nutrition and reduce food insecurity.

## 2. Materials and Methods

### 2.1. Study Population and Data Collection

This study was part of a broader evaluation of changes in school meal operations and uptake following DC-M in Maryland Community School meal programs [25]. Families were recruited from summer meal sites operated by community schools, particularly those that had been newly adopted or were eligible for CEP. Data collection occurred in two phases, which were Wave 1, during the operation of pandemic waivers and before DC-M implementation in early fall 2022, and Wave 2, one year after the expiration of pandemic waivers and DC-M implementation in early fall 2023, enabling a comparative analysis of program and policy effects over time. This study was approved by the Johns Hopkins School of Public Health Institutional Review Board and the Prince George’s County Public Schools, Division of Accountability, Department of Testing, Research and Evaluation.

A cross-sectional survey in English or Spanish was administered in-person to household heads aged 18 years or older with one or more children attending a Prince George’s County Public Schools district. One representative per household completed the questionnaire using a personal device or a study-owned tablet, in-person, with assistance provided when necessary. The survey was administered via Qualtrics (https://www.qualtrics.com; accessed 10 June 2025). Participants were given a small gift as an incentive for completing the survey. The survey collected data on household food security status, participation in food assistance programs, and child dietary habits. Diet quality was assessed using the Dietary Screener Questionnaire (DSQ), while food security was measured with the USDA’s Six-Item Short Form. Data from both waves were integrated for a comprehensive analysis.

### 2.2. Sociodemographic Characteristics and Participation in Food Assistance Programs

Self-reported sociodemographic characteristics, including race, ethnicity, number of children in the household, parental education level, and participation in food assistance programs, were included in the survey. For analytical purposes, the number of children in the household was dichotomized as 0–1 or ≥2 children, and parental education level was grouped into ≤high school (including less than 9th grade, high school without a diploma, and high school with a diploma or General Educational Diploma) or ≥some college (including some college and college degree or more). Participation in food assistance programs (including SNAP, summer SNAP, WIC, the Pandemic Electronic Benefit Transfer (P-EBT) Program, and Prince George’s County Public Schools summer meal program) the past summer was classified as “Yes” or combined as “No/Don’t know” responses to maintain adequate sample size in subgroups for analysis. To address multicollinearity in regression models, participation in P-EBT or summer SNAP was grouped into a composite variable, “summer programs.” Participants with more than one child were instructed to answer child-specific questions for the child with the next upcoming birthday. Child ethnicity (Hispanic or non-Hispanic), sex (male or female), and age (2–8 years or 9–18 years) were categorized based on parental reports.

### 2.3. Assessment of Housing Stability and Food Security

Housing stability was assessed using a single yes or no question: “Are you worried that in the next 2 months, you may not have stable housing?” Food security status was evaluated using the USDA’s Six-Item Short Form, with households categorized into high, low, or very low food security following USDA guidelines [26].

### 2.4. Estimation of Dietary Intake and Evaluation of Diet Quality

The DSQ evaluates child dietary patterns across seven components: fiber, calcium, whole grains, sugar, dairy, fruits, and vegetables. Daily dietary intake was estimated from the DSQ using established procedures from the National Cancer Institute Epidemiology and Genomics Research Program [27]. Due to the DSQ’s limitations in capturing energy intake, traditional diet quality indices, such as the Healthy Eating Index (HEI) and the Dietary Approaches to Stop Hypertension Index (DASHI), which require total caloric data, could not be computed. Instead, diet quality was assessed by comparing estimated intakes against sex- and age-specific daily nutritional goals from the Dietary Guidelines [28], as detailed in Supplemental Table S1. Percent differences from these recommendations for each dietary component were calculated to quantify deviations. For recommendations based on energy intake, values from the proposed Calorie Level Assessment criteria were used [28].

A composite score based on seven dietary components was developed to quantify overall diet quality. Specifically, percent differences from dietary recommendations for each component were stratified into quartiles (Q1–Q4), with Q4 representing the largest deviation. The composite score summed Q4 occurrences across all seven components, resulting in a score ranging from 0 to 7. Higher scores indicate poorer diet quality. For analytic purposes, scores were categorized into higher diet quality (≤1) and lower diet quality (≥2). They were used as a binary dependent variable in subsequent regression analyses.

### 2.5. Statistical Analysis

Variables were stratified as follows for descriptive analyses: household characteristics by the wave of data collection, ethnicity, food security status, and summer meal participation; child characteristics and food security status by the wave of data collection and ethnicity for descriptive analysis; and dietary outcomes by the wave of data collection, ethnicity, sex, age, and summer meal participation. Chi-squared and Fisher’s exact tests were used to examine differences in categorical variables, while the Wilcoxon–Mann–Whitney test was used for non-normally distributed continuous variables. Covariates with ≤7% missing values were handled using mode imputation. Bar graphs illustrate food security status, while radar plots visualize percent differences from dietary recommendations for each dietary component.

Four multivariate logistic models were created to explore significant determinants of diet quality in the study sample:

- Model 1: Key predictors (food security status and summer meal participation) + sociodemographic variables (ethnicity, age, sex, parental education level, number of children in the household, and housing stability).
- Model 2: Key predictors + participation in food assistance programs other than summer meals (SNAP, WIC, and summer programs).
- Model 3: Key predictors + temporal trends (wave of data collection).
- Model 4: Full model integrating all covariates.

Additionally, stepwise regression was performed to identify a model with optimal fit (the lowest AIC). Results from all models were presented as adjusted odds ratios (exponentiated coefficients) representing the likelihood of poorer diet quality (overall diet quality score ≥ 2) relative to the reference group. All analyses used R (version 4.2.1), with statistical significance defined as *p* < 0.05.

## 3. Results

A total of 158 households were included in the analysis (n = 101 during Wave 1 and n = 57 during Wave 2), with 135 providing complete dietary data (Supplementary Figure S1). Key findings revealed significant ethnic disparities in participation in food assistance programs and food security status and changes in dietary patterns between waves of data collection.

### 3.1. Sociodemographic Characteristics

As shown in Table 1, the total sample comprised 53.16% Hispanic participants and 46.84% non-Hispanic participants (of whom 35.44% were Black or African American, 7.59% were White, and 3.80% were Native Hawaiian, Pacific Islander, or others). The majority of participants reported an education level of high school or lower (57.59%) and having ≥2 children in their household (70.25%). Sociodemographic characteristics were comparable between Wave 1 and Wave 2 participants. However, a greater proportion of Hispanic participants reported an education level of high school or lower (79.76% vs. 32.43%, *p* < 0.001) and a household size of two or more children (79.76% vs. 59.46%, *p* < 0.001) compared to non-Hispanic participants.

### 3.2. Dietary Behaviors and Food Acquisition

The majority of households (56.77%) reported sharing >5 meals together per week. The predominant food source was in-store purchases (84.18%), followed by restaurant or carry-out (17.09%), food pantries or free meal programs (16.46%), and school food programs (15.19%). Wave 2 participants had higher utilization rates of restaurants or carry-out (26.32% vs. 11.88%, *p* = 0.021), school food programs (21.05% vs. 11.88%, *p* = 0.123), and food pantries or other free meal programs (22.81% vs. 12.87%, *p* = 0.106) than Wave 1 participants. Food shopping and preparation were predominantly conducted by one or both parents.

### 3.3. Food Assistance Program Participation

Surveyed households had the highest participation in the summer meal program (67.72%), followed by summer programs (45.57%), SNAP (41.14%), and WIC (27.22%) (Table 1). Participants in Wave 2 had significantly higher participation in SNAP (50.88% vs. 35.64%, *p* = 0.039) and summer programs (57.89% vs. 38.61%, *p* = 0.011) than those in Wave 1. Ethnicity-stratified analysis revealed significantly higher participation in WIC among Hispanic participants compared to non-Hispanic participants (42.86% vs. 9.46%, *p* < 0.001). However, this pattern was not observed for other food assistance programs.

### 3.4. Summer Meal Participation

Summer meal participation reached 32.28% in the total sample, with 60.13% reporting non-participation and 7.59% reporting uncertainty about participation over the past summer (Table 1). Wave 2 exhibited significantly higher participation rates than Wave 1 (43.86% vs. 25.74%, *p* = 0.019), and Hispanic participants showed higher participation rates than non-Hispanic participants (40.48% vs. 22.97%, *p* = 0.019).

Among summer meal participants, engagement in concurrent summer programs was notably higher than that of non-participants (57.50% vs. 37.89%, *p* = 0.036) (Supplemental Table S2). However, no significant differences were found between summer meal participants and non-participants regarding other characteristics.

Frequency of utilization among summer meal participants was primarily daily (35.29%) or several times per week (37.25%). Though not statistically significant, summer meal utilization was more frequent in Wave 2 (44.00% daily) than in Wave 1 (26.92% daily). Transportation to meal sites was primarily by car or walking (37.25% each), with most participants reporting travel times < 15 min (60.78%). The primary barriers to participation included lack of awareness (53.68%), personal choice (20.00%), and transportation or location issues (11.58%).

Meal satisfaction analysis revealed that 37.25% of participants rated breakfast and 15.69% rated lunch as comparable to home meals. Positive feedback (including descriptions like “good,” “healthy,” “variety,” “fresh”) was reported by 15.69% of participants for breakfast and 19.61% for lunch. Additionally, Wave 2 participants reported higher satisfaction rates compared to Wave 1, with fewer participants reporting differences between the provided meals and home meals (breakfast: 4.00% vs. 23.08%; lunch: 8.00% vs. 26.92%).

### 3.5. Housing Instability

Nearly one-third of households (29.11%) experienced housing instability, with a marginally higher prevalence among Wave 1 participants compared to Wave 2 (30.69% vs. 26.31%, *p* = 0.138) (Table 1). Although Hispanic participants reported higher rates of housing instability compared to non-Hispanic participants (33.33% vs. 24.32%), this difference was not significant (*p* = 0.262).

### 3.6. Food Insecurity

**Figure 1:**
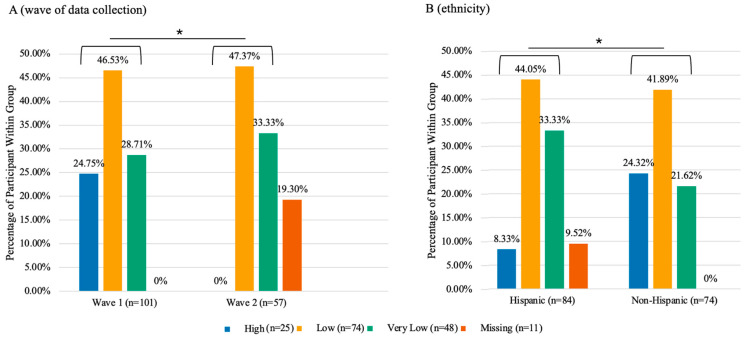
Differences in food security status by wave of data collection and ethnicity in the total sample (n = 158). This chart compares food security status across two waves of data collection (**A**) and between Hispanic and non-Hispanic groups (**B**). Differences were assessed using Fisher’s exact tests. Missing values were excluded from the contingency table when computing the statistical test (* *p* < 0.05).

Food security deteriorated significantly between waves, as illustrated in Figure 1. Households with high food security dropped from 24.75% in Wave 1 to 0% in Wave 2 (*p* < 0.001). Food insecurity disproportionally affected Hispanic households, which exhibited significantly lower rates of high food security (8.33% vs. 24.32%, *p* = 0.020).

A subgroup analysis comparing households with varying levels of food security showed that those experiencing very low food security had a significantly higher proportion of unstable housing compared to households with low to high food security (40.48% vs. 23.66%, *p* = 0.046) (Supplemental Table S2). No other significant differences were observed.

### 3.7. Child Diet Quality

**Figure 2:**
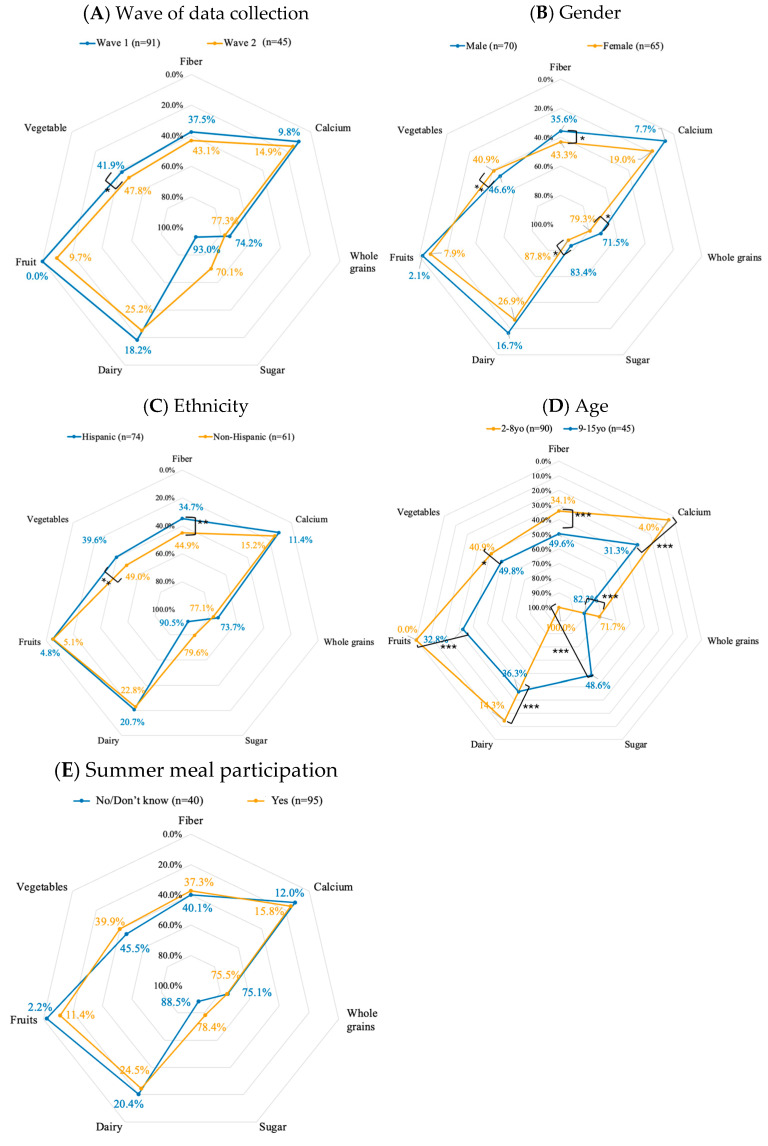
Estimated percent difference from recommended intake by wave of data collection, sex, ethnicity, age, and summer meal participation in the subset sample (n = 135). The radar plot displays percentage differences from recommended intakes for fiber, calcium, whole grains, sugar, dairy, vegetables, and fruits. Differences were assessed using the Wilcoxon–Mann–Whitney test. Values closer to 0% (or dots closer to the outermost border) indicate closer alignment with recommended intake levels (better intake). (**A**) Children in Wave 1, (**C**) from Hispanic households, (**D**) or of younger age demonstrated better intake of most dietary components, except sugar. (**B**) Boys had better intake of most dietary components, except vegetables. (**E**) Summer meal participation was associated with better intake of vegetables, fiber, sugar, and whole grains, though insignificant. (* *p* < 0.05, ** *p* < 0.01, *** *p* < 0.001).

The diet quality analysis included 135 children (n = 91 during Wave 1 and n = 44 during Wave 2), comprising 90 children aged 2–8 years and 45 children aged 9–18 years (Table 2). Among these children, 51.85% were male and 48.15% were female. No significant differences were observed in the overall diet quality score when stratified by wave of data collection (*p* = 0.886) or ethnicity (*p* = 0.077). The median diet quality score remained constant (=1) across both ethnic groups and waves. However, changes were observed in individual food components, including fiber, calcium, whole grains, sugar, dairy, fruit, and vegetables, as shown in Figure 2.

During the second wave of data collection, children exhibited poorer intake across most food components, with the exception of sugar (Figure 2A). Specifically, a statistically significant difference in vegetable consumption was observed in Wave 2 compared to Wave 1 (41.9% vs. 47.8%, *p* = 0.046).

In collapsing all data across waves, sex differences were evident in dietary patterns (Figure 2B). Boys showed better intake across most dietary components, except for vegetables. These differences were significant for fiber (*p* = 0.049), whole grains (*p* = 0.011), and sugar consumption (*p* = 0.036). Conversely, girls demonstrated significantly better vegetable consumption (*p* = 0.009). Ethnicity-stratified analysis revealed that Hispanic children had better intake across most dietary components, with the exception of sugar (Figure 2C). They demonstrated significantly better intake of fiber (*p* = 0.002) and vegetables (*p* = 0.002) compared to non-Hispanic participants. Age-stratified analysis (2–8 years vs. 9–18 years) revealed significant differences across all dietary components (Figure 2D). Older children consistently showed worse intake across all dietary components except sugar, with strong statistical significance for most dietary components (*p* < 0.001) and vegetable consumption (*p* = 0.012). No significant differences in dietary components were observed when stratified by food security status or participation in the summer meal program (Figure 2E).

### 3.8. Determinants of Diet Quality Among Children of Surveyed Participants

A series of logistic regression models was constructed to examine factors associated with poor overall diet quality in the subset sample (n = 135), collapsing all data across waves. When only sociodemographic characteristics were considered (Table 3, Model 1), older age (OR = 39.86, *p* < 0.01) and very low food security (OR = 7.15, *p* = 0.035), but not low food security (OR = 4.52, *p* = 0.079), were associated with significantly higher odds of poor diet quality. Conversely, participation in the summer meal program was associated with significantly lower odds of poor diet quality (OR = 0.22, *p* = 0.019).

Model 2, which incorporated food assistance program participation, identified participation in the summer meal program as the only significant predictor (OR = 0.39, *p* = 0.045), while food security status was no longer significant. Model 3, which examined wave-specific effects, did not identify any significant covariates.

The full model (Model 4) confirmed the significance of age, very low food security, and summer meal participation. Older children demonstrated substantially higher odds of poor diet quality (OR = 52.97, *p* < 0.001), as did those experiencing very low food security (OR = 8.42, *p* = 0.036). Participation in the summer meal program remained a potentially protective factor (OR = 0.23, *p* = 0.030). Additionally, Hispanic ethnicity emerged as a significant potentially protective factor (OR = 0.17, *p* = 0.019).

**Figure 3:**
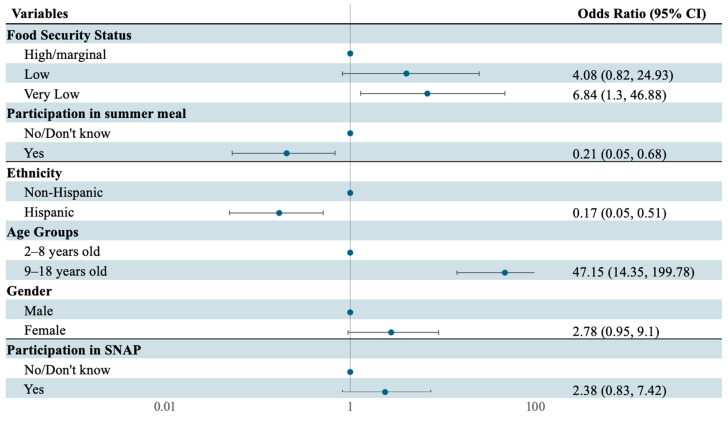
Significant determinants of diet quality among children in the final stepwise model (n = 135). The forest plot visualizes adjusted odds ratios (exponentiated coefficients) with 95% confidence intervals from the final stepwise model, representing the likelihood of poorer diet quality (overall diet quality score ≥ 2) relative to the reference group. Very low food security and older age (9–18 years) had significantly increased odds of poor diet quality. Summer meal participation and Hispanic household status suggested possible protective effects. Model adjusted for housing stability, concurrent food assistance, and demographic covariates. CI, confidence interval.

The final stepwise model selected food security status, participation in the summer meal program, ethnicity, age, sex, and participation in SNAP (Figure 3). This model revealed that older age (OR = 47.15, *p* < 0.001) and very low food security (OR = 6.84, *p* = 0.033) were associated with significantly higher odds of poor diet quality. Potentially protective factors included participation in the summer meal program (OR = 0.21, *p* = 0.015) and Hispanic ethnicity (OR = 0.17, *p* = 0.003).

## 4. Discussion

The large difference in food security among summer meal participants from Wave 1 and Wave 2 (24.75% and 0%, respectively) signals a critical time for U.S. child nutrition policy. This study demonstrates that even effective programs like the summer meal program face systemic barriers, such as transportation inequities and outreach failures. By dissecting dietary, demographic, and programmatic data from 158 low-income households, we propose strategies to transform these initiatives into robust tools for breaking intergenerational cycles of food insecurity.

### 4.1. Summer Meal Participation

Our analysis identified the summer meal program as a critical but underutilized intervention. Participation in the summer meal program was positively associated with improved diet quality, reinforcing the program’s value in supporting children’s nutritional needs during school closures [29]. Notably, Hispanic households demonstrated nearly double the participation rates of non-Hispanic households, potentially driven by larger household sizes and heightened food insecurity, suggesting greater reliance on supplemental nutrition programs. Nevertheless, existing food assistance programs remained insufficient to address the prevalent food insecurity in these communities.

### 4.2. Food Insecurity

Alarmingly high rates of food insecurity were observed among surveyed households, with significant ethnic disparities—Hispanic households experienced disproportionately higher food insecurity compared to non-Hispanic households. Of particular concern, food security changed markedly from Wave 1 to Wave 2, despite greater participation in the summer meal program, with the proportion of households reporting high food security dropping from 24.75% to 0%. This difference indicates compounding stressors, possibly from economic and environmental factors, that warrant urgent investigation, although causative factors were not directly assessed in this study.

Paradoxically, children from Hispanic households demonstrated better nutritional outcomes, likely attributable to their higher engagement with WIC (42.86% in Hispanic households vs. 9.46% in non-Hispanic households) and the summer meal program. The WIC food package included fruits, vegetables, and whole grains, with studies showing improved diet quality among participants, particularly from increased fruit and vegetable consumption [30,31]. Similarly, Zimmerman et al. found that summer meals significantly contribute to daily nutritional goals [29]. While program participation likely contributes to this potentially protective effect, our quantitative methodology cannot establish causal pathways between cultural practices and dietary resilience. Tovar et al. suggested that culturally mediated factors, such as traditional diets rich in legumes, whole grains, and fresh produce, alongside Latinx caregivers modeling healthy eating and prioritizing nutrition education, may enhance resilience [32]. Future research is needed to understand the influence of cultural identity on diet quality and food strategies under economic constraints.

### 4.3. Barriers and Potential Solutions

Despite increased participation rates in Wave 2—potentially attributable to DC-M implementation—38.60% of eligible households in Wave 2 remained either non-participants or uncertain about their enrollment status, underscoring systemic implementation gaps. Two interrelated systemic barriers were identified as primary obstacles to program efficacy. (1) Nearly 50% of non-participants lacked awareness of the program or their eligibility, suggesting critical outreach deficiencies. (2) Transportation or location challenges disproportionately impacted rural communities and urban neighborhoods lacking reliable public transit. These findings align with earlier analyses by the Food Research and Action Center (2017) [33] emphasizing persistent geographic and informational inequities.

Addressing these barriers requires a multilayered approach that combines program expansion and targeted outreach. First, developing linguistically and culturally tailored outreach campaigns delivered through trusted community channels, particularly those serving immigrant populations, would help bridge awareness gaps. Second, reinstating flexibility for mobile meal distribution or subsidizing public transit, particularly in high-need or remote regions, may alleviate transportation and geographic barriers. Mobile distribution systems lowered operational costs and allowed for decentralized services during the pandemic [15,23]. Adopting a similar strategy would increase the number of service sites and extend the program’s reach. Additionally, improved transit would enhance the accessibility of service sites, increasing the frequency of summer meal utilization.

Given the potentially protective interplay of summer meal participation and the challenges of low participation, we recommend leveraging community liaisons within the community school model in Maryland to connect families with available programs. Community liaisons can ensure clear and consistent communication with families, identify nearby meal sites, and address logistical barriers. This approach aligns with the community school framework and has the potential to close nutritional gaps by increasing participation in food assistance programs.

### 4.4. Diet Quality

Children in Wave 2 exhibited significantly poorer overall diet quality than those in Wave 1, particularly in vegetable consumption. This decline was not attributable to age differences, because older children (9–18 years) consistently demonstrated poorer dietary intake across both time points, but Wave 1 had a higher proportion of older children. Additionally, dietary analysis uncovered concerning patterns of insufficient whole grain consumption and excessive sugar intake among surveyed children. If this pattern is consistent across broader populations, future nutritional interventions must prioritize strategies to reduce sugar consumption while promoting whole grain intake. However, evidence from other regions is lacking.

Consistent with previous studies [34], households experiencing very low food security had eight-fold higher odds of poor child diet quality, highlighting the devastating nutritional consequences of food insecurity. These results emphasize the need for dual-focused interventions addressing immediate food access and systemic socioeconomic drivers.

Composite diet quality scores, such as the Healthy Eating Index (HEI), are widely used to assess diet quality in alignment with the Dietary Guidelines for Americans, utilizing adequacy and moderation components to evaluate adherence to dietary recommendations and enabling standardized comparisons across populations [35,36,37]. Similarly to our study, composite scores have been adapted in previous research to align with the available data or specific populations. For example, HEI-Toddlers-2020 was developed for young children [38], and a modified HEI was tailored to hemodialysis-specific nutritional guidelines [39], both using the same adequacy and moderation metrics. These adaptations demonstrate the flexibility of composite scoring methods in evaluating diet quality among populations with unique dietary behaviors or nutritional needs.

### 4.5. Age and Sex Differences in Diet Quality

This study observed age-related differences in diet quality consistent with findings from previous studies [28,40,41]. Increasing food autonomy and shift to family diets with increasing age may increase consumption of energy-dense, nutrient-poor foods while reducing intake of dairy, grains, fruits, and vegetables [42].

Gender disparities revealed complex sociocultural influences. Deslippe et al. explored the influence of sex on dietary behaviors and preferences. They found that sex significantly affects how peer, family, and school environments shape children’s eating habits as they transition into adolescence [43]. For example, girls socialize during lunch, which is primarily sedentary, while boys socialize through team sports. Additionally, girls are more driven by external motivators, such as healthy eating and traditional body norms, whereas boys are more motivated by internal factors, such as autonomy and physical performance [43]. Therefore, researchers should consider a sex- and age-targeted approach to improve the effectiveness of nutrition interventions. Further research is needed to better understand the underlying motivations informing food choices in low-income children of different ages and sexes.

### 4.6. Strengths and Limitations

The strengths of this study include the validity of survey responses ensured by bot protection, reliable assessments of food insecurity and diet through validated questionnaires (USDA’s Six-Item Short Form and DSQ), response accuracy enhanced by assistance with survey completion, and reliable results from regression models accounting for other food assistance programs, food insecurity, and housing instability.

The study’s limitations include its limited generalizability beyond students at community schools with summer meal programs in Prince George’s County, Maryland. Data from other regions are needed for broader applicability and confirmation of our findings. Second, the convenience sampling approach—recruiting participants from existing summer meal program sites—may limit the representativeness of the sample. Families who chose to participate might differ systematically from non-participants due to selection bias. Consequently, the findings may disproportionately reflect families already engaged with food assistance services rather than all eligible households. Third, the cross-sectional design prevents causal inference between observed factors and diet quality. Fourth, our small sample size restricts statistical power, especially for subgroup analyses. Unusually high odds ratios (such as for older children) likely reflect small subgroup sizes rather than true effects.

According to the U.S. Census Bureau’s Supplemental Poverty Measure, 41% of low-income children were Hispanic, 28.1% were non-Hispanic White, 20.8% were non-Hispanic Black, 5.6% were Asian, and 4.3% were American Indian in 2022 [44]. While racially diverse (see Section 3.1), our sample (n = 158) overrepresents Hispanic participants and underrepresents non-Hispanic White children compared to the national trend. Additionally, the Prince George’s County Public Schools district in Maryland comprises a racially diverse student population, with 12% White, 60% Black, 20% Hispanic, 4% Asian, and 4% identifying as other races [45]. Our sample is not intended to be representative of the population in the district, and it does not mirror the demographic distribution of children or households in the county.

Additionally, the DSQ, while validated for assessing intake of specific food groups, does not capture total energy intake. Existing algorithms and methodologies do not support the calculation of total calorie intake from DSQ responses. This limitation precluded the use of established diet quality indices, such as HEI and DASHI, which require caloric data for computation. Consequently, our study relied on a modified HEI composite score, comparing dietary intake to sex- and age-specific guidelines. Key food groups, such as protein, were not assessed in this study, and the use of questionnaires introduces the potential for reporting bias or seasonal variability.

## 5. Conclusions

Our findings highlight the importance of summer meal programs in addressing food insecurity, aligning with the existing literature, which underscores their critical role in supporting children’s nutritional needs during school breaks. Adequate funding for such programs is essential to ensure their effectiveness and reach. While our results provide valuable insights for informing policies related to the NSLP and other federal nutrition initiatives, the small sample size limits broader applications. Additional research with larger, more representative samples is needed to strengthen the evidence base and guide policy development more effectively.

Studies that monitor and evaluate nutritional outcomes of existing federal nutrition programs, such as this one, are critical for identifying actionable pathways to improve program efficacy and equity, particularly as policymakers refine initiatives like SUN Bucks and SFSP. Future studies should prioritize longitudinal assessments of summer meals’ dietary impacts, cost–benefit analyses of alternative distribution systems, and ethnographic investigations into cultural adaptation strategies for diverse populations. Policymakers can transform summer nutrition programs into powerful tools for reducing dietary disparities—a crucial step toward breaking the intergenerational cycle of food-insecurity-related health consequences.

## Figures and Tables

**Table 1:** Household sociodemographic characteristics and participation in food assistance programs by wave of data collection and ethnicity in the total sample (n = 158).

Characteristics	Total Sample	Wave 1	Wave 2		Hispanic	Non-Hispanic	
n (%)	158 (100)	101 (100)	57 (100)	*p*-Value ^a^	84 (100)	74 (100)	*p*-Value ^b^
Ethnicity				0.153			
Non-Hispanic	74 (46.84)	43 (42.57)	31 (54.39)		/	/	
Hispanic	84 (53.16)	58 (57.43)	26 (45.61)		/	/	
Education				0.307			<0.001
≤ high school	91 (57.59)	61 (60.40)	30 (52.63)		67 (79.76)	24 (32.43)	
≥ some college	66 (41.77)	39 (38.61)	27 (47.37)		16 (19.05)	50 (67.57)	
Missing	1 (0.64)	1 (0.99)	0 (0)		1 (1.19)	0 (0)	
Number of children in the household				0.370			<0.001
0–1 child	41 (25.95)	28 (27.72)	13 (22.81)		12 (14.29)	29 (39.19)	
≥2 children	111 (70.25)	67 (66.34)	44 (77.19)		67 (79.76)	44 (59.46)	
Missing	6 (3.80)	6 (5.94)	0 (0)		5 (5.95)	1 (1.35)	
Housing stability				0.493			0.262
Stable	104 (65.82)	64 (63.37)	40 (70.18)		53 (63.10)	51 (68.92)	
Not stable	46 (29.12)	31 (30.69)	15 (26.31)		28 (33.33)	18 (24.32)	
Missing	8 (5.06)	6 (5.94)	2 (3.51)		3 (3.57)	5 (6.76)	
Food security status				<0.001			0.020
High/marginal	25 (15.82)	25 (24.75)	0 (0.00)		7 (8.33)	18 (24.32)	
Low	74 (46.84)	47 (46.53)	27 (47.37)		38 (45.24)	36 (48.66)	
Very low	48 (30.38)	29 (28.72)	19 (33.33)		30 (35.72)	18 (24.32)	
Missing	11 (6.96)	0 (0.00)	11 (19.30)		9 (10.71)	2 (2.70)	
Summer meal				0.019			0.019
No/don’t know	107 (67.72)	75 (74.26)	32 (56.14)		50 (59.52)	57 (77.03)	
Yes	51 (32.28)	26 (25.74)	25 (43.86)		34 (40.48)	17 (22.97)	
SNAP				0.039			0.193
No/don’t know	91 (57.59)	65 (64.36)	26 (45.61)		45 (53.57)	46 (62.16)	
Yes	65 (41.14)	36 (35.64)	29 (50.88)		39 (46.43)	26 (35.14)	
Missing	2 (1.27)	0 (0)	2 (3.51)		0 (0)	2 (2.70)	
Summer programs				0.011			0.941
No/don’t know	84 (53.16)	62 (61.39)	22 (38.60)		45 (53.57)	39 (52.71)	
Yes	72 (45.57)	39 (38.61)	33 (57.89)		39 (46.43)	33 (44.59)	
Missing	2 (1.27)	0 (0)	2 (3.51)		0 (0)	2 (2.70)	
WIC				0.150			<0.001
No/don’t know	113 (71.52)	77 (76.24)	36 (63.16)		48 (57.14)	65 (87.84)	
Yes	43 (27.21)	24 (23.76)	19 (33.33)		36 (42.86)	7 (9.46)	
Missing	2 (1.27)	0 (0)	2 (3.51)		0 (0)	2 (2.70)	

^a^ *p*-value for difference between Wave 1 and Wave 2 using Chi-squared or Fisher’s exact test. ^b^ *p*-value for difference between Hispanic and non-Hispanic using Chi-squared or Fisher’s exact test. Missing values were excluded from the contingency table when computing the statistical test.

**Table 2:** Child characteristics and diet quality by wave of data collection and ethnicity in the subset sample (n = 135).

Characteristics	Total Sample	Wave 1	Wave 2		Hispanic	Non-Hispanic	
n (%)	135 (100)	91 (100)	44 (100)	*p*-Value ^a^	61 (100)	74 (100)	*p*-Value ^b^
Age Groups				0.299			0.903
2–8 years	90 (66.67)	58 (63.74)	32 (72.73)		41 (67.21)	49 (66.22)	
9–18 years	45 (33.33)	33 (36.26)	12 (27.27)		20 (32.79)	25 (33.78)	
Sex				0.765			0.898
Male	70 (51.85)	48 (52.75)	22 (50.00)		32 (52.46)	38 (51.35)	
Female	65 (48.15)	43 (47.25)	22 (50.00)		29 (47.54)	36 (48.65)	
Overall Diet Score				0.886			0.077
High-quality (≤1)	84 (62.22)	57 (62.64)	27 (61.36)		33 (54.10)	51 (68.92)	
Low-quality (≥2)	51 (37.78)	34 (37.36)	17 (38.64)		28 (45.90)	23 (31.08)	

^a^ *p*-value for difference between Wave 1 and Wave 2 using Chi-squared or Fisher’s exact test. ^b^ *p*-value for difference between Hispanic and non-Hispanic using Chi-squared or Fisher’s exact test.

**Table 3:** Summary of odds ratios across four models analyzing food security and summer meal participation (n = 135).

Variables	Model 1	Model 2	Model 3	Model 4
Food security status				
High/marginal	Reference group	Reference group	Reference group	Reference group
Low	4.52	1.68	2.00	4.74
Very Low	7.15 *	2.14	2.45	8.42 *
Participation in summer meal				
No/don’t know	Reference group	Reference group	Reference group	Reference group
Yes	0.22 *	0.39 *	0.45	0.23 *
Ethnicity				
Non-Hispanic	Reference group	/	/	Reference group
Hispanic	0.22	/	/	0.17 *
Age groups				
2–8 years old	Reference group	/	/	Reference group
9–18 years old	39.86 **	/	/	52.97 ***
Gender				
Male	Reference group	/	/	Reference group
Female	2.54	/	/	2.95
Education				
≤ high school	Reference group	/	/	Reference group
≥ some college	1.33	/	/	1.56
Number of children				
0–1	Reference group	/	/	Reference group
≥2	0.86	/	/	0.95
Housing stability				
Stable	Reference group	/	/	Reference group
Not stable	1.50	/	/	1.56
Participation in SNAP			/	
No/don’t know	/	Reference group	/	Reference group
Yes	/	0.90		2.84
Participation in WIC			/	
No/don’t know	/	Reference group	/	Reference group
Yes	/	0.77		0.84
Participation in summer programs			/	
No/don’t know	/	Reference group	/	Reference group
Yes	/	2.07		0.86
Wave				
1	/	/	Reference group	Reference group
2	/	/	0.85	0.61

* *p* < 0.05; ** *p* < 0.01; *** *p* < 0.001; Model 1: Key predictors (food security status and summer meal participation) + sociodemographic variables (ethnicity, age, sex, parental education level, number of children in the household, and housing stability); Model 2: Key predictors + participation in food assistance programs other than summer meals (SNAP, WIC, and summer programs); Model 3: Key predictors + temporal trends (wave of data collection); Model 4: Full model integrating all covariates.

## Data Availability

Deidentified data may be available from the corresponding author upon request due to concerns related to privacy and ethical issues.

## Supplementary Materials

The following supporting information can be downloaded at https://www.mdpi.com/article/10.3390/nu17132055/s1, Figure S1: Flow chart of the study population; Table S1: Recommended intake of dietary components by age and gender; Table S2: Household characteristics by summer meal participation and food security status in the subset sample (n = 135).

## Abbreviations

The following abbreviations are used in this manuscript:

AIC	Akaike Information Criterion
CEP	Community Eligibility Provision
DASHI	Dietary Approaches to Stop Hypertension Index
DC-M	Direct Certification through Medicaid
DSQ	Dietary Screener Questionnaire
HEI	Healthy Eating Index
NSLP	National School Lunch Program
P-EBT	Pandemic Electronic Benefit Transfer
SBP	School Breakfast Program
SFSP	Summer Food Service Program
SNAP	Supplemental Nutrition Assistance Program
SSO	Seamless Summer Option (NSLP summer extension)
SUN Bucks	Summer Electronic Benefit Transfer (Summer EBT)
WIC	Special Supplemental Nutrition Program for Women, Infants, and Children
