# Supplementary material for: The Interplay Between Summer Meals, Food Insecurity, and Diet Quality Among Low-Income Children in Maryland, USA: A Multiphase Cross-Sectional Study
Source: Nutrients. 2025 Jun 20;17(13):2055. doi: 10.3390/nu17132055 (PMC12251470; doi:10.3390/nu17132055)
Supplement: Supplementary file 1 [file nutrients-17-02055-s001.zip › nutrients-3672630-supplementary.pdf]

Supplemental Table S1. Recommended intake of dietary components by age and gender.

	Age (years) and Sex						
	2-4		5-8		9-13		14-18
**Dietary Components**	Male	Female	Male	Female	Male	Female	Male
Fiber (gm)	≥14.0	≥14.0	≥17.0	≥20.0	≥22.0	≥25.0	≥31.0
Calcium (mg)	700.0	700.0	1000.0	1000.0	1300.0	1300.0	1300.0
Added sugar (tsp equiv.)	≤6.0	≤6.0	≤8.4	≤7.2	≤10.8	≤9.6	≤13.2
Whole grain (ounce equiv.)	1.5-3.0	1.5-2.5	2.0-3.0	2.0-3.0	3.0-4.5	2.5-3.5	3.0-5.0
Dairy (cup equiv.)	2.0-2.5	2.0-2.5	2.5-2.5	2.5-2.5	3.0-3.0	3.0-3.0	3.0-3.0
Vegetables (cup equiv.)	1.0-2.0	1.0-1.5	1.5-2.5	1.5-2.5	2.0-3.5	1.5-3.0	2.5-4.0
Fruits (cup equiv.)	1.0-1.5	1.0-1.5	1.0-2.0	1.0-1.5	1.5-2.0	1.5-2.0	2.0-2.5

Recommendation adopted from the Dietary Guidelines for Americans 2020-2025 [28].

Supplemental Figure S1. Flow chart of the study population.

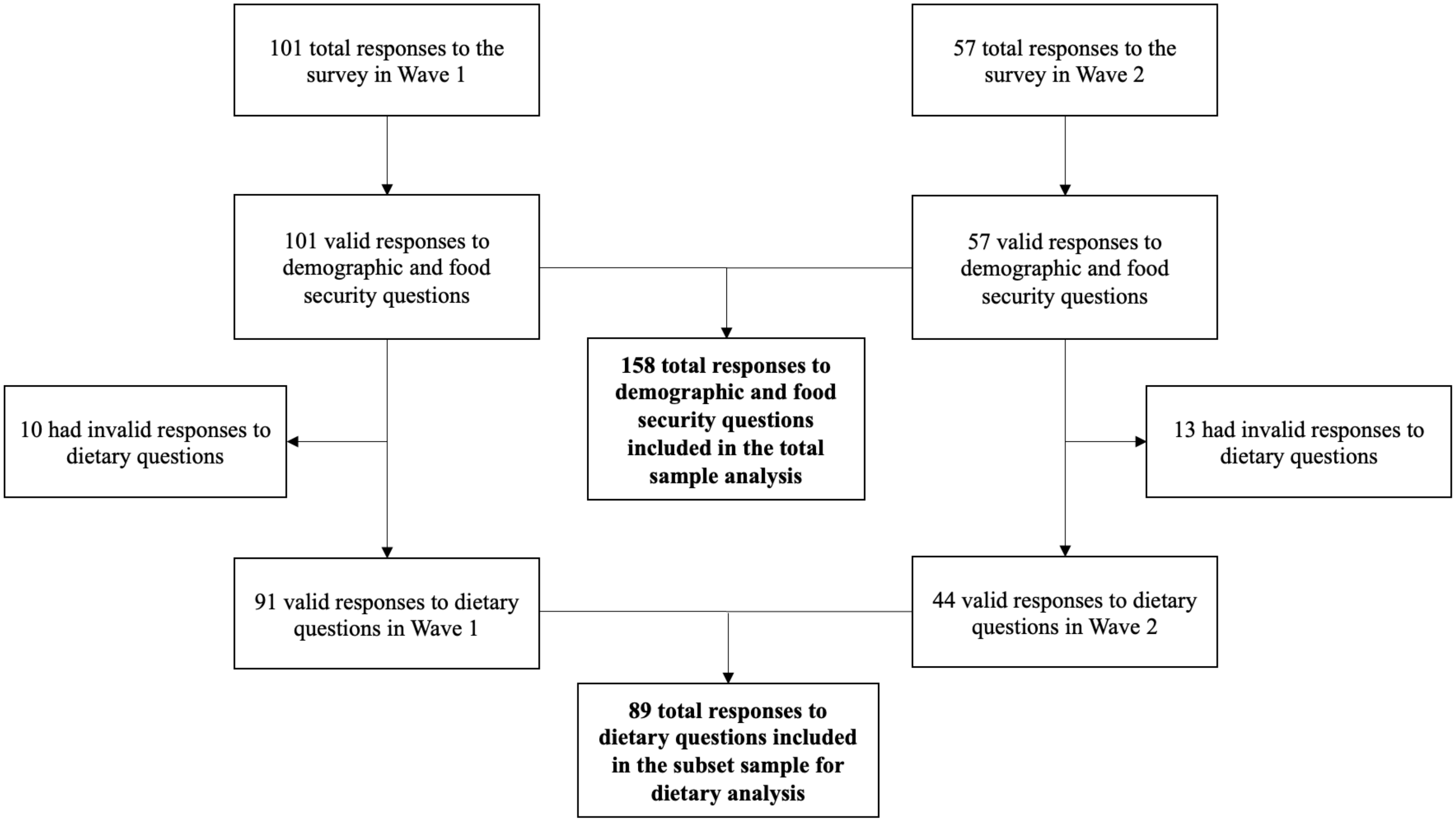

Supplemental Table S2. Household characteristics by summer meal participation and food security status in the subset sample (n=135).

Characteristics n (%)	Summer Meal Participants 40 (100)	Summer Meal non-Participants 95 (100)	*p*-value[a]	Very Low Food Security 42 (100)	Low to High Food Security 93 (100)	*p*-value[b]
**Ethnicity**			0.055			0.222
Non-Hispanic	13 (32.50)	48 (50.53)		16 (38.10)	45 (48.39)	
Hispanic	27 (67.50)	47 (49.47)		26 (61.90)	48 (51.61)	
**Education**			0.321			0.095
≤ High school	26 (65.00)	53 (55.79)		29 (69.05)	50 (53.76)	
≥ Some college	14 (35.00)	42 (44.21)		13 (30.95)	43 (46.24)	
**Household size**			0.086			0.805
0-1 child	6 (15.00)	28 (29.47)		10 (23.81)	24 (25.81)	
≥ 2 children	34 (85.00)	67 (70.53)		32 (76.19)	69 (74.19)	
**Food security status**			0.141			/
High or marginal	3 (8.33)	20 (21.98)		/	/	
Low	22 (50.00)	48 (48.35)		/	/	
Very low	15 (41.67)	27 (29.67)		/	/	
**Housing stability**			0.817			0.046
Stable housing	29 (72.50)	67 (70.53)		25 (59.52)	71 (76.34)	
Unstable housing	11 (27.50)	28 (29.47)		17 (40.48)	22 (23.66)	
**Wave of data collection**			0.001			0.603
1 (early fall 2022)	19 (47.50)	72 (75.79)		27 (64.29)	64 (75.29)	
2 (early fall 2023)	21 (52.50)	23 (24.21)		15 (35.71)	29 (24.71)	
**Participation in summer meal**			/			0.298
No/Don't know	/	/		27 (64.29)	68 (73.12)	
Yes	/	/		15 (35.71)	25 (26.88)	
**Participation in SNAP**			0.071			0.475
No/Don't know	19 (47.50)	61 (64.21)		23 (54.76)	57 (61.29)	
Yes	21 (52.50)	34 (35.79)		19 (45.24)	36 (38.71)	
**Participation in WIC**			0.064			0.106
No/Don't know	26 (65.00)	76 (80.00)		28 (66.67)	74 (79.57)	
Yes	14 (35.00)	19 (20.00)		14 (33.33)	19 (20.43)	
**Participation in summer programs**			0.036			0.538
No/Don't know	17 (42.5)	59 (62.11)		22 (52.38)	54 (58.06)	

Yes	23 (57.5)	36 (37.89)	20 (47.62)	39 (41.194)

[a] *p*-value for difference between summer meal participants vs. summer meal non-participants.
[b] *p*-value for difference between household with very low food security vs. low to high food security.
